# Ventilation

**Published:** 1889-07

**Authors:** 


### VENTILATION.

Perhaps, few who have heard of the "Black Hole of Calcuta," know the terrible facts that have rendered the place famous and made it the synonym of all that is to be dreaded from foul air and over-crowding.

At eight o'clock on the evening of June 20, 1756, one hundred and forty-six prisoners, officers and men, black and white, and of different nationalities, were thrust into a room, eighteen feet square—with two windows on one of the four sides, heavily barred with iron—giving to each inmate forty cubic feet of space. In ten hours one hundred and twenty-three were found dead—only twenty-three being alive!

Another instance is where, in 1742, the High Constable of Westminster, London, committed twenty-eight persons to prison, where they were thrust by the keeper into a hole six feet square and five feet ten inches high—the windows being close shut. In a very short time four of the inmates were suffocated. These facts show the poisonous effects of the human breath—or of respired air. Prof. Brown-Sequard has recently made some experiments that are not only highly interesting, but show why the expired air of man and animals is so deadly. From the condensed vapor of the expired air he produced a liquid so poisonous that when injected beneath the skin of rabbits it produced almost instant death. This poison he found to be not a microbe, but an alkaloid. His conclusions are that the expired air of all animals contains a poison more fatal than carbonic acid.

It is well for the people to understand these facts. They cry aloud for better ventilation and purer air—for less crowding in home and church, and hall and school-room.—*Board Health Bulletin* (Iowa)

